# The Spatiotemporal Evolution of Ecosystem Health and Its Driving Factors in Karst Rocky Desertification Areas A Case Study of Guizhou Province, China

**DOI:** 10.1002/ece3.71374

**Published:** 2025-05-25

**Authors:** Beibei Zhang, Zhongfa Zhou

**Affiliations:** ^1^ School of Karst Science/School of Geography and Environment Science Guizhou Normal University Guiyang China; ^2^ State Engineering Technology Institute for Karst Desertification Control Guiyang China; ^3^ Guizhou Forestry School/Guizhou Vocational College of Ecological Energy Guiyang China

**Keywords:** driving factors, ecosystem health, GTWR, karst desertification areas, PSR

## Abstract

Understanding the health status and drivers of ecosystems in karst desertification areas is crucial for sustainable development. Using the “Pressure‐State‐Response (PSR)” framework, this study assessed the ecosystem health levels in Guizhou Province, a typical karst region, for the years 2000, 2005, 2010, 2015, and 2020, and identified the main drivers affecting ecosystem health levels using the Spatiotemporal Geographically Weighted Regression (GTWR) model. The results show: (1) During the study period, the ecosystem health level in Guizhou Province was predominantly rated as excellent or relatively excellent, showing a positive development trend with an overall upward trajectory. The value increased from 0.534 in 2000 to 0.622 in 2020. Areas with better ecosystem health were mainly distributed in southern Zunyi, eastern Guiyang, and northern Qiannan Prefecture. (2) Spatially, the Ecosystem Health Index (EHI) at the county level in Guizhou Province showed significant positive spatial correlation at the 99.9% confidence level, with “H‐H clustering” mainly in the northern part of Qiannan and the southern part of Zunyi. (3) Different factors have varying impacts on the ecosystem health in the study area. Precipitation, relative humidity, and nighttime lights mainly have a positive driving effect on ecosystem health in Guizhou Province, while temperature, vegetation coverage, and land use intensity are the dominant factors with a negative driving effect on ecosystem health. The findings can provide a theoretical basis for formulating ecological protection and sustainable development policies in karst desertification regions.

## Graphical abstract

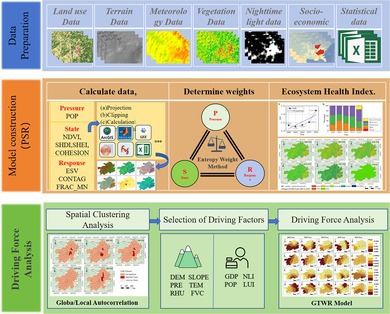

## 1 Introduction

Ecosystem health refers to the ability of an ecosystem to maintain a relatively stable and good state in its dynamic processes, manifested as the ecosystem's capacity for self‐regulation, self‐repair, and self‐renewal. (Wu et al. 2021). In the past few decades, human activities have increasingly disturbed ecosystems. (Li et al. 2021), This has led to the degradation of at least two‐thirds of the ecosystems on Earth, with ecosystem health becoming an increasingly prominent issue. This poses a serious threat to the sustainable development of human society and the economy. (Wang, Yao, et al. 2024). Therefore, evaluating the health of regional ecosystems and identifying their main driving forces is of great significance for ecosystem management, protection, and restoration. (Ford et al. 2015). Common methods for measuring ecosystem health mainly include the bioindicator method and the indicator system method (Fu et al. 2021; Wang et al. 2022), These two methods dominate the relevant literature, accounting for 88.3% and 11.7%, respectively. The bioindicator method is closely related to biology and involves selecting key species or indicator communities within the ecosystem as indicators to assess the health status of the ecosystem (Liu et al. 2023). The bioindicator method is mainly applied to aquatic ecosystems (Cui et al. 2024). For example, fish, algae, and waterbirds can be used as bioindicators for assessing wetland health. This approach is applicable to evaluations of specific natural ecosystem types, yet requires substantial field monitoring data. It mainly assesses the ecosystem's internal health conditions while paying limited attention to external environmental influences (Liao et al. 2024). The indicator system method involves constructing an evaluation system for different types of ecosystems, covering multiple aspects of both the ecological and social environments. This method can better reflect the characteristics and processes of complex socio‐ecological systems. A common indicator system method is the Vitality‐Organization‐Resilience (VOR) model (Gu et al. 2020; Yadav et al. 2024), Pressure‐State‐Response (PSR) Model, Analytic Hierarchy Process (AHP) (Bai et al. 2023; Kim et al. 2021), Entropy weight method (EWM) (Wang, Wang, et al. 2024), Fuzzy Mathematical Methods (FMM)and Set Pair Analysis (SPA) (Mandal et al. 2024). Among them, the VOR model and the PSR model are the most widely applied. As research deepens, scholars have gradually expanded the connotations of the models. For example, by adding driving forces (D) and impacts (I) to the PSR model, the Driving‐Pressure‐State‐Impact‐Response (DPSIR) model is formed to more comprehensively explain ecosystem health. The elements of ecosystem services have also been incorporated into the VOR model, forming the Vitality‐Organization‐Resilience‐Service (VORS) model, thereby further perfecting the evaluation system (Chen et al. 2024). Furthermore, the evaluation system has been enhanced by techniques such as the Technique for Order Preference by Similarity to Ideal Solution (TOPSIS) (Xu et al. 2019; Zeng et al. 2016; Zhang et al. 2022), Backpropagation Neural Network (BPNN) (Wang et al. 2017), and Genetic Algorithm‐optimized Backpropagation Artificial Neural Network (GA‐BPANN) (Xu et al. 2019). However, existing studies have yet to establish a unified indicator system. Much of the research has focused on ecosystem health characteristics, health level classification, and regional disparities (Ding‐Zhao et al. 2024; Meng et al. 2023). Previous studies have primarily concentrated on large cities, urban agglomerations, and developed regions, with limited investigation into ecosystem health in karst rocky desertification areas—particularly regarding the spatiotemporal differentiation of driving factors.

China is the country with the largest area and widest distribution of carbonate rock karst, with about 200 million people living in karst rocky desertification areas, accounting for approximately 10% (Bai et al. 2023) of the national economy. Especially in the southwestern region of China, centered around Guizhou, which is located in the upper reaches of the Yangtze and Pearl Rivers, the economy is underdeveloped, and ecological issues such as soil erosion and rocky desertification are constraining the coordinated development of ecological security and socio‐economic progress in southern China (Wu 2015). Karst ecosystems deliver diverse ecosystem services, encompassing climate regulation, water retention, soil formation and conservation, biodiversity preservation, and agricultural provisioning (Zhao, Cheng, et al. 2021; Jiao et al. 2022). Therefore, assessing the spatial differentiation of karst ecosystem services is an inevitable choice for addressing ecological issues and ensuring the security of ecosystem service provision. It plays a significant role in promoting regional sustainable development (Cheng et al. 2022; Wu and Zhang 2023).

**FIGURE 1:**
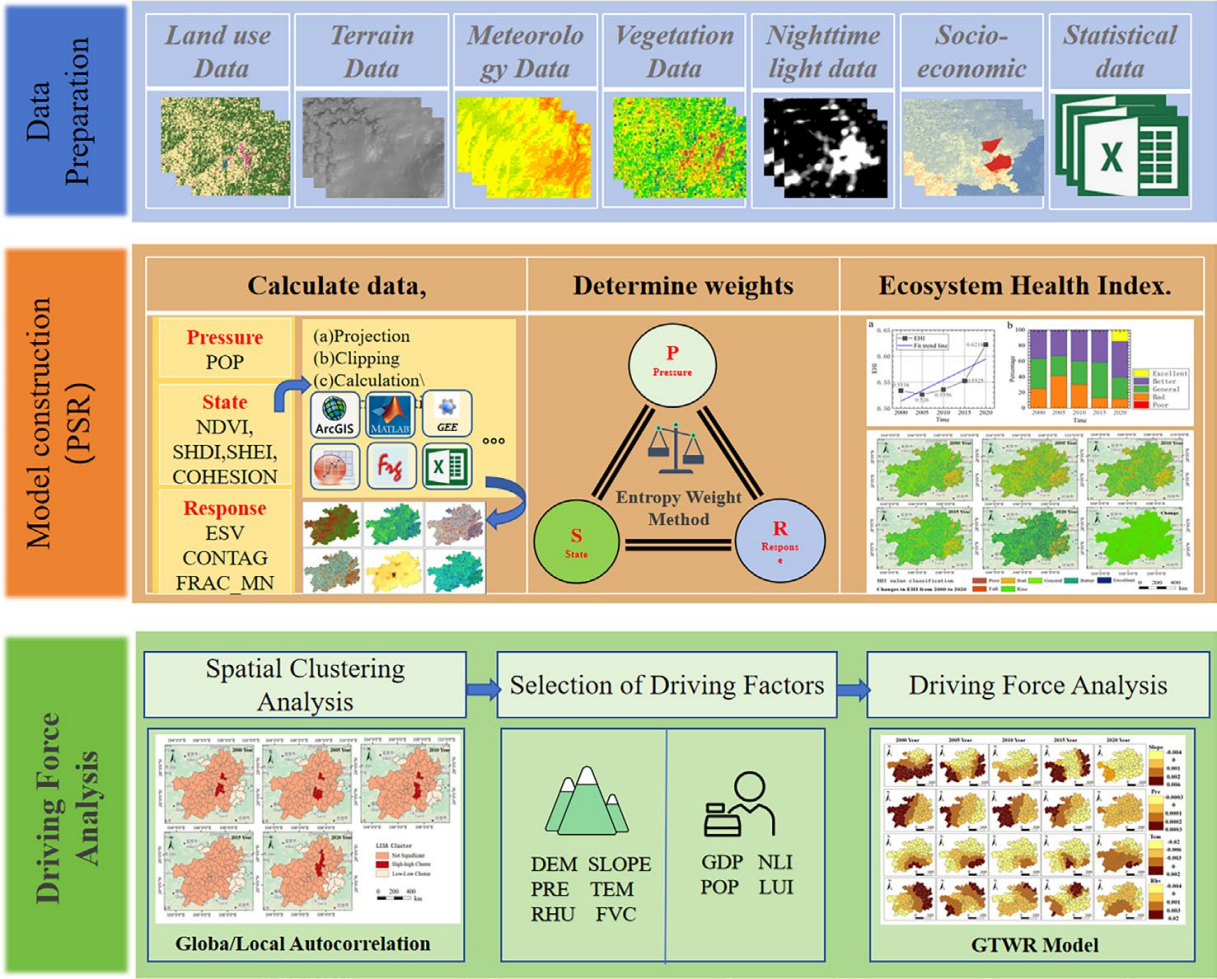
Research process flowchart.

In light of this, this study establishes an ecosystem health assessment system for karst rocky desertification areas by integrating the Pressure‐State‐Response (PSR) model and entropy weight method, based on regional characteristics. The framework is applied to evaluate the ecosystem health dynamics in Guizhou Province from 2000 to 2020. Subsequently, spatial autocorrelation analysis is employed to examine the aggregation patterns of ecosystem health status. Finally, the Geographically and Temporally Weighted Regression (GTWR) model is utilized to investigate the spatiotemporal heterogeneity of key driving factors affecting ecosystem health. These approaches provide policymakers with targeted insights for ecological restoration strategies, offering both theoretical foundations and empirical support for sustainable ecosystem management in Guizhou Province. The specific research framework and process of this article are shown in Figure 1.

## 2 Study Area Overview

**FIGURE 2:**
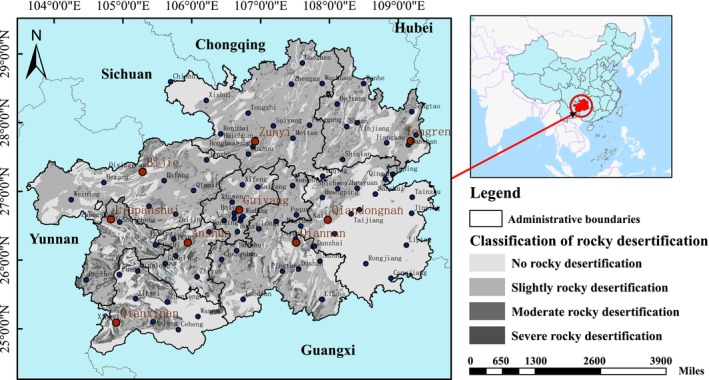
Map of the study area.

Guizhou is located in the southwestern region of China (103°36′ to 109°35′ E, 24°37′ to 29°13′N), bordered by Hunan Province to the east, Guangxi Zhuang Autonomous Region to the south, Yunnan Province to the west, and Chongqing Municipality and Sichuan Province to the north. It has a total area of 176,167 km^2^, accounting for 1.8% of the country's total land area. The terrain is high in the west and low in the east, with an average elevation of 1100 m. The province is characterized by undulating mountains, complex geomorphological types, and unique natural landscapes. Guizhou is located in the southwestern region of China (Ding‐Zhao et al. 2024), with a diverse climate that is typically around 15°C in average annual temperature for most areas. It is one of the most typical regions for karst development in the world (Na et al. 2023), with karst exposure accounting for 61.9% of the province's total area. The climate is warm and humid, with excellent water and thermal conditions. The favorable ecological environment makes it an important ecological barrier for the upper reaches of the Yangtze and Pearl Rivers (He and Tang 2023). Despite its fragile ecological environment, Guizhou has made certain achievements in ecological protection and has a good ecological foundation, becoming one of the first national ecological civilization pilot zones. Guizhou is known for its beautiful mountains and rivers, pleasant climate, diverse ethnic groups, rich resources, and huge development potential. Its unique geographical environment has created the beauty and magic of Guizhou, making it an ideal summer retreat. Guizhou is the first national demonstration zone for the aggregation and development of big data, with a focus on building a big data base centered around Guiyang, Yili, and the Gui'an New Area. It is an inland province close to coastal ports and the Yangtze River and is one of the 12 provinces (autonomous regions, municipalities) implementing the Western Development Strategy in China. In 2023, Guizhou's gross domestic product reached 2091.325 billion yuan, with a per capita regional gross domestic product of 54,172 yuan. By the end of the year, the permanent population of the province was 38.65 million, and the urbanization rate was 55.94%. The spatial distribution map of the study area is shown in Figure 2.

## 3 Materials and Methods

### 3.1 Data Sources

#### 3.1.1 Ecosystem Health Assessment Data

Ecosystem health assessment data includes land use data, population distribution data, vegetation cover data, and socio‐economic data, etc. The land use and population distribution data are derived from the Resource and Environment Science Data Center of the Chinese Academy of Sciences; vegetation cover data is sourced from the MODIS series remote sensing data from 2001 to 2020 in the GEE (Google Earth Engine) platform database; socio‐economic data comes from statistical yearbooks of various provinces and cities, as well as the “National Compilation of Agricultural Product Cost and Benefit Data” (Table 1).

**TABLE 1:** Research Data Sources and Description.

Data name	Format	Resolution	Data source
Land Use Data (Lucc)	tif	30 m	Resources and Environmental Science Data Center, Chinese Academy of Sciences (https://www.resdc.cn/)
Population Data (pop)	tif	1 km	
GDP	tif	1 km	
Digital Elevation Mode (DEM)	tif	90 m	Geospatial Data Cloud (https://www.gscloud.cn/)
Slope	tif	90 m	Derived from DEM calculations
Monthly Average Temperature Data (T)	.nc	1 km	National Tibetan Plateau/Third Pole Environment Data Center (https://data.tpdc.ac.cn/home)
Monthly Precipitation Data (P)	.nc	1 km	
Monthly Atmospheric Humidity Index Data (RHU)	.nc	1 km	
Vegetation Cover (FVC)	tif	500 m	Googleearthengine (https://developers.google.cn/earth‐engine/)
Night Lights Data (NLI)	tif	500	National Oceanic and Atmospheric Administration (https://www.fisheries.noaa.gov/)
Land Use Intensity (LUI)	tif	1 km	Derived from Land Use Data
Socioeconomic Data	excel	—	Statistical Yearbook of Each Province and City

#### 3.1.2 Ecosystem Health Driving Factors Data

Ecosystem health driving factors include elevation (digital elevation model, DEM), slope (SLPOE), annual mean precipitation (precipitation, PRE), annual mean temperature (temperature, TEM), annual mean relative humidity (relative humidity, RHU), gross domestic product (GDP), vegetation coverage (fraction of vegetation coverage, FVC), nighttime light intensity (nighttime light intensity, NLI), and land use intensity (land use intensity, LUI). Among them, DEM data comes from the Geospatial Data Cloud, and SLPOE data is derived from DEM data; PRE, TEM, and RHU data come from the National Tibetan Plateau/Third Pole Environment Data Center; GDP data comes from the Resources and Environmental Science Data Center, Chinese Academy of Sciences; FVC data comes from the Google Earth Engine platform; NLI data comes from the National Oceanic and Atmospheric Administration (NOAA). For specific descriptions, see Table 1. All data were uniformly projected to the WGS_1984_UTM_Zone_47N coordinate system using ArcGIS 10.8 software, resampled to 1 km spatial resolution raster data for calculation, and statistically analyzed at the county level.

### 3.2 Ecosystem Health Assessment

#### 3.2.1 Pressure‐State‐Response Framework

The Pressure‐State‐Response (PSR) framework is based on the causal relationships among pressure, state, and response, and was initially proposed by the Organization for Economic Cooperation and Development (OECD) (Levrel et al. 2009). The framework not only describes the pressures that human activities exert on ecosystems, altering their vitality, organization, functions, and resilience, but also assesses human responses to these changes (Das et al. 2020; Li, Liu, et al. 2024), thereby restoring or improving the health of the ecosystem (Sun et al. 2019). In this context, the pressure indicators represent the stress imposed on ecosystems by natural production and human activities. The population, which signifies the pressure from human activities, is selected as an indicator for evaluating pressure. State indicators represent the ecological environment status and changes in the study area (Xiao et al. 2024), Considering the biological characteristics and ecological functions of the ecological environment, four indicators are selected as state evaluation indicators: Normalized Difference Vegetation Index (NDVI), Shannon's Diversity Index (SHDI), Shannon's Evenness Index (SHEI), and Cohesion Index (COHESION) (Ge et al. 2022; Wu et al. 2022; Zhao, Han, et al. 2021). Among them, NDVI is related to vegetation growth and net primary production. SHDI and SHEI characterize landscape diversity and stability, while COHESION represents landscape connectivity. Response indicators are used to measure the effectiveness of human measures taken for ecological restoration. Ecosystem Services Value (ESV), as an important criterion for evaluating ecological service functions, has been widely defined as a key indicator for assessing ecosystem health. The Contagion Index (CONTAG) can reflect the integrity of the landscape, and the Mean Fractal Dimension Index (FRAC_MN) is used to measure the complexity of the landscape (Ren et al. 2022). Therefore, ESV, CONTAG, and FRAC_MN are selected as response layer indicators to construct a framework for assessing the health of ecosystems in karst ecologically fragile areas. In this context, SHDI, SHEI, COHESION, CONTAG, and FRAC_MN are calculated using the landscape index calculation software Fragstats 4.2. For specific introductions and calculation methods, please refer to Table 2. The calculation of ESV refers to the equivalent coefficient table for ESV per unit area of terrestrial ecosystems in China proposed by Xie Gaodi and others (Gaodi et al. 2015). By categorizing according to different land use types, the ESV for the study area for the years 2000, 2005, 2010, 2015, and 2020 is obtained. The selection of these parameters as indicators for assessing ecosystem health in the study area was primarily based on the following criteria: First, each indicator has clear ecological significance and can systematically characterize key attributes of karst ecosystems (e.g., productivity, diversity, connectivity). Second, all indicators have been widely validated in academic research and demonstrate reliable application foundations in karst ecosystem studies. Third, standardized calculation methods exist for these indicators (using Fragstats software for landscape metrics and Xie Gaodi's equivalent coefficient method for ESV), ensuring result comparability. Finally, these indicators comprehensively cover all evaluation dimensions of the PSR framework, reflecting both natural ecological characteristics and human intervention effects, while being highly sensitive to rocky desertification processes—making them particularly suitable for health assessment in vulnerable karst ecosystems.

**TABLE 2:** Ecosystem Health Assessment Indicator System and Calculation Methods.

Criterion layer	Criterion layer weight	Indicator layer	Indicator attribute	Calculation method	Parameter meaning	Indicator layer weight
Pressure layer	0.1301	POP	Negative	Total Population of All Regions	—	0.1301
State layer	0.4969	NDVI	Positive	NDVI = (NIR − R)/(NIR + *R*)	NIR stands for Near‐Infrared band reflectance, and R stands for Infrared band reflectance	0.1277
		SHDI	Positive	SHDI = $-\sum_{i=1}^{m}\left(p_{i}\times \text{In}\;p_{i}\right)$	$P_{i}$ represents the proportion of the area of the i‐th type of patch to the total area of the landscape. m represents the number of landscape types.	0.1184
		SHEI	Positive	SHEI = $-\sum_{i=1}^{m}\left(p_{i}\right)\ln p_{i}/\ln m)$	asabove	0.1257
		COHESION	Positive	COHESION = $\left[1-\frac{\sum_{i=1}^{m}\sum_{j=1}^{n}P_{\mathrm{ij}}}{\sum_{i=1}^{m}\sum_{j=1}^{n}P_{\mathrm{ij}}\sqrt{a_{\mathrm{ij}}}}\right]\times {\left[1-\frac{1}{\sqrt{n}}\right]}^{-1}\times 100$	$P_{\mathrm{ij}}$ represents the perimeter (in meters) of patch ij, $a_{\mathrm{ij}}$ epresents the area (in square meters) of patch ij, and N indicates the total number of landscape patches.	0.1251
Response layer	0.373	ESV	Positive	ESV = $\sum_{i=1}^{n}\left(A_{i}V_{\mathrm{ci}}\right)$	$A_{i}$ epresents the area of the k‐th land use type in the study area, $V_{\mathrm{ci}}$ represents the coefficient of ecosystem service value.	0.1229
		CONTAG	Positive	CONTAG = $\left[1+\frac{\sum_{i=1}^{m}\sum_{j=1}^{n}P_{i}\frac{g_{\mathrm{ik}}}{\sum_{i=1}^{m}g_{\mathrm{ik}}}}{2\ln m}\right]\times 100$	$P_{i}$ has the same meaning as above, $g_{\mathrm{ik}}$ represents the connectivity number, mrepresents the total number of patch types in the landscape	0.1275
		FRAC_MN	Positive	FRAC_MN = $\sum_{i=1}^{m}\sum_{j=1}^{n}2\ln\left(\frac{0.25P_{\mathrm{ij}}}{\ln a_{\mathrm{ij}}}\right)/N$	$P_{\mathrm{ij}},a_{\mathrm{ij}}$ and N have the same meanings as previously mentioned.	0.1226

#### 3.2.2 Entropy Weight Method

The normalization of each index is carried out using the Min‐Max method, scaling the data of each index to the range of 0 to 1 (Cui et al. 2018; Xu et al. 2014). The specific calculation formula is as follows:

For positive indicators:

(1) $$Y_{\mathrm{ij}}=\frac{\left(x_{\mathrm{ij}}-x_{\text{imin}}\right)}{\left(x_{\text{imax}}-x_{\text{imin}}\right)}$$

For negative indicators:

(2) $$Y_{\mathrm{ij}}=\frac{\left(x_{\text{imin}}-x_{\mathrm{ij}}\right)}{\left(x_{\text{imax}}-x_{\text{imin}}\right)}$$

In the formula, $Y_{\mathrm{ij}}$ represents the standardized index value, $x_{\mathrm{ij}}$ represents the initial value of the index, $x_{\text{imax}}$ and $x_{\text{imin}}$ represent the maximum and minimum values of the index, i represents the number of indices, taking values from 1 to 8, and j represents the year.

The weight of the evaluation indicators is determined using the entropy weight method, which is not subject to subjective influences and, based on relevant teaching theories, can reflect the utility information of each indicator. The specific calculation formula is as follows:

The calculation formula for the entropy value ($E_{i}$) is as follows:

(3) $$E_{i}=-k\sum_{j=1}^{n}f_{\mathrm{ij}}\ln f_{\mathrm{ij}}$$

In the formula, k = 1 / lnn. 当 $f_{\mathrm{ij}}$ = 0时, $f_{\mathrm{ij}}\ln f_{\mathrm{ij}}$ = 0.

1. The calculation formula for the difference coefficient ($d_{i}$) is as follows:

(4) $$d_{i}=1-E_{i}\left(i=1,2\ldots ,m\right)$$

2 The calculation formula for the weight of the indicator ($w_{i}$)is as follows:

(5) $$w_{i}=\frac{d_{i}}{\sum_{j=1}^{n}r_{\mathrm{ij}}}$$

In the formula, $0\leq w_{i}\leq 1$, $\sum_{j=1}^{n}w_{i}=1$, The weights of the indicators calculated using this method are shown in Table 1.

The comprehensive evaluation index is calculated by summing the standardized values of each evaluation indicator weighted by their respective weights.

(6) $${\mathrm{EHI}}_{i}=\sum_{\mathrm{ij}-1}^{n}w_{i}\times Y_{\mathrm{ij}}$$

In the formula, ${\mathrm{EHI}}_{i}$ represents the ecosystem health index for the i‐th year.

At present, there is no unified standard for the classification of EHI. Referring to existing research findings and the actual situation in Guizhou Province, the EHI calculation results for county‐level areas in Guizhou are divided into four grades: EHI ∈ (0, 0.5) is bad (Level I), EHI ∈ (0.5, 0.55) is general (Level II), EHI ∈ (0.55, 0.6) is better (Level III), EHI ∈ (0.6, 1) is excellent (Level IV). Moreover, since the EHI consistently showed an increasing trend throughout the study period in the research area, the EHI change values were categorized into three levels: Level I (0.06 < ΔEHI < 0.08), Level II (0.08 < ΔEHI < 0.1), and Level III (0.1 < ΔEHI < 0.13).

### 3.3 Spatial Autocorrelation Analysis

Spatial autocorrelation analysis is used to measure the correlation and difference of a certain observation value in adjacent spaces, which is divided into global autocorrelation and local autocorrelation. Among them, global autocorrelation is used to analyze the strength of the correlation of the observed variable, and is characterized by the global Moran's I (IG), which ranges from [−1,1] (Ren et al. 2024); Local spatial autocorrelation analysis is used to measure the strength of the association and heterogeneity of local unit autocorrelation, and is characterized by the local Moran's I (IL) (Das et al. 2021; He et al. 2022). The specific formula is as follows:

(7) $$I_{G}=\frac{n\sum_{i=1}^{n}\sum_{j=1}^{n}W_{\mathrm{ij}}}{\sum_{i=1}^{n}\sum_{j=1}^{n}W_{\mathrm{ij}}\sum_{i=1}^{n}{\left(x_{i}-\bar{x}\right)}^{2}}$$

(8) $$I_{L}=\frac{\sum_{j=1,j+i}^{n}W_{\mathrm{ij}}\left(\left(x_{i}-\bar{x}\right)\right)\left(\left(x_{i}-\bar{x}\right)\right)}{\sum_{i=1}^{n}{\left(x_{i}-\bar{x}\right)}^{2}}$$

In the formula, n represents the number of counties, $\bar{x}$ represents the average value of the observed values, $x_{i},\;x_{j}$ represent the observed values of i and j, $W_{\mathrm{ij}}$ represents the spatial weight between i and j.

### 3.4 Spatio‐Temporal Geographically Weighted Regression

In this paper, the GTWR model is adopted to explore the spatiotemporal characteristics of the driving factors of ecosystem health in the Loess Plateau. (Huang et al. 2010; Ran et al. 2023; Zhang et al. 2024), The formula is as follows:

(9) $$Y_{i}=\beta_{0}\left(u_{i},v_{i},t_{i}\right)+\sum_{l=1}^{k}\beta_{l}\left(u_{i},v_{i},t_{i}\right)X_{\mathrm{il}}+\delta_{i}$$

In the formula, *Yi* is the observed variable, *ui*, *vi* and *ti* are the longitude, latitude, and time, respectively, *βl* (*ui*, *vi*, *ti*) is the regression coefficient for the l‐th independent variable of the i‐th element, *Xil* is the explanatory variable, and *δi* is the residual term.

In the context of driving factors, the calculation formula for LUI:

(10) $$\mathrm{LUI}=\sum_{i=0}^{n}A_{i}S_{i}/S_{c}$$

In the formula, *Ai* represents the intensity grade of the i‐th land use type, with the intensity grades for various land use types being as follows: construction land (4), arable land (3), grassland (2.5), forest land (2), water bodies (2), and unused land (1); *Si* represents the area of the i‐th land use type, and *S*c represents the total land area.

## 4 Results and Analysis

### 4.1 Spatiotemporal Evolution Characteristics of Ecosystem Health in Guizhou Province

**FIGURE 3:**
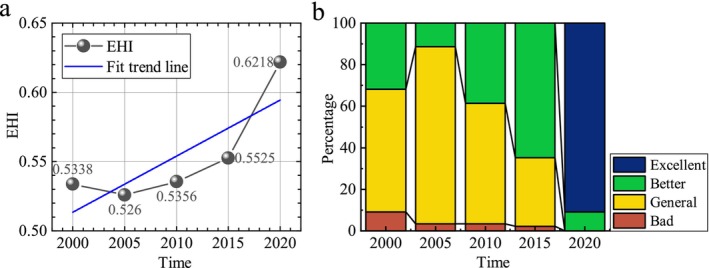
Shows the trend of ecosystem health (EHI) changes in Guizhou Province from 2000 to 2020 (a) and the proportion of different EHI levels (b).

Figure 3a shows the average EHI values for Guizhou Province in the years 2000, 2005, 2010, 2015, and 2020 to be 0.5338, 0.526, 0.5356, 0.5525, and 0.6218, respectively, overall presenting a fluctuating upward trend. Among them, the EHI average in 2005 was the lowest, which may be related to multiple natural disasters that occurred in Guizhou Province in 2005, such as landslides and collapses in Guiyang, Zunyi's Honghuagang District, Duyun area, Bijie region, Tongren region, and other places. Looking at the proportion of different levels of EHI, The area with EHI rated as good (Grade III) and relatively good (Grade IV) showed an upward trend. In Guizhou Province, the ecosystem health level was primarily classified as relatively good (Grade III), increasing from 31.8% in 2000 to 64.8% in 2015. The proportion of areas with EHI rated as excellent (Grade IV) rose from 0% in 2000 to 90.9% in 2020. Meanwhile, the area with EHI rated as poor (Grade I) and fair (Grade II) exhibited a declining trend. Among these, the area with poor ecosystem health in Guizhou Province was the smallest, decreasing from 9% in 2000 to 0% in 2020. Regions with fair ecosystem health levels were mainly converted into areas with relatively good ecosystem health levels. By 2020, approximately 90% of the areas in Guizhou Province had achieved a relatively good ecosystem health level, as shown in Figure 1b. Over the past two decades, the ecosystem health level in Guizhou Province is mainly dominated by the best and better grades, with a good development trend and a gradual upward trend.

**FIGURE 4:**
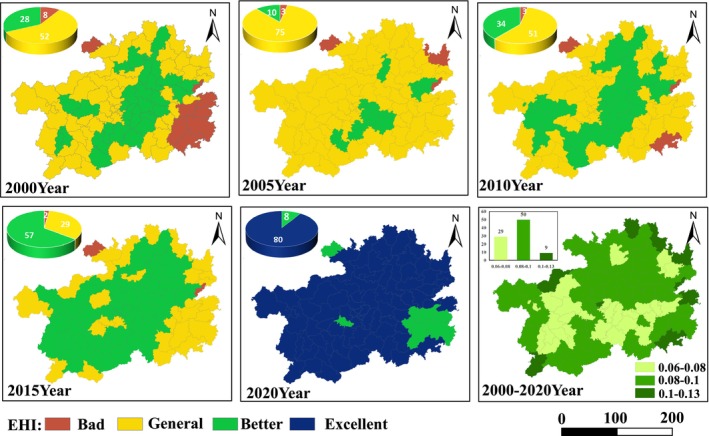
Spatial distribution and spatiotemporal changes of ecosystem health in Guizhou province.

Spatially, the ecosystem health levels in Guizhou Province exhibit spatial heterogeneity (Figure 4). Overall, the ecosystem health level in the central region was higher than that in the peripheral areas. From 2000 to 2015, the total area with “excellent” ecosystem health gradually expanded, primarily distributed in southern Zunyi, eastern Guiyang, and northern Qiannan Prefecture. This improvement was largely attributed to ecological protection and restoration measures, which significantly enhanced the ecosystem health in these regions. In 2005, the area with “excellent” ecosystem health was the smallest, accounting for only 11.4% of Guizhou Province's total area and mainly concentrated in northern Qiannan Prefecture. By 2020, the ecosystem health level in Guizhou Province reached its peak, with approximately 90.9% of the region classified as “relatively good” or better. Areas with relatively poor ecosystem health were primarily located in the eastern and southern parts of Qiandongnan Prefecture. This may be due to the region's transitional terrain between the Yunnan‐Guizhou Plateau and the Guangxi hills, characterized by complex topography, intertwined mountains, hills, and valleys, as well as steep slopes that make the soil prone to erosion. Additionally, the area experiences variable weather patterns and concentrated rainfall, which can trigger natural disasters such as landslides and debris flows, further exacerbating ecological fragility and contributing to lower health levels. Other areas with relatively low ecosystem health included Chishui City in northwestern Zunyi and northeastern Tongren. Overall, Guizhou Province maintained a relatively high level of ecosystem health across all periods. Compared to 2000, the province saw an increase in ecosystem health ranging between 0.08 and 0.13 by 2020, with the largest proportion (50%) falling within the 0.08–0.1 range. The fastest‐growing regions were mainly distributed in the eastern part of Guizhou Province.

### 4.2 Spatial Aggregation Characteristics of Ecosystem Health in Guizhou Province

The results of the global spatial autocorrelation Moran's I index analysis are shown in Table 3. The global Moran's I indices for EHI in Guizhou Province in the years 2000, 2005, 2010, 2015, and 2020 are 0.241, 0.2, 0.239, 0.373, and 0.315, respectively. The z‐values are all greater than the critical value of 2.58 at the significance level test, and all passed the significance level test with *p* < 0.001. This indicates that there is a significant positive spatial autocorrelation of EHI at the county level in Guizhou Province at a 99.9% confidence level. That is, areas with higher EHI tend to be spatially adjacent to areas with higher EHI, and areas with lower EHI tend to be spatially adjacent to areas with lower EHI.

**TABLE 3:** Global Autocorrelation Test of EHI.

Year	Moran's I index	*z*‐score	*p*
2000	0.241	3.6266	< 0.001
2005	0.200	3.1910	< 0.001
2010	0.239	3.7911	< 0.001
2015	0.373	5.8436	< 0.001
2020	0.315	4.9379	< 0.001

**FIGURE 5:**
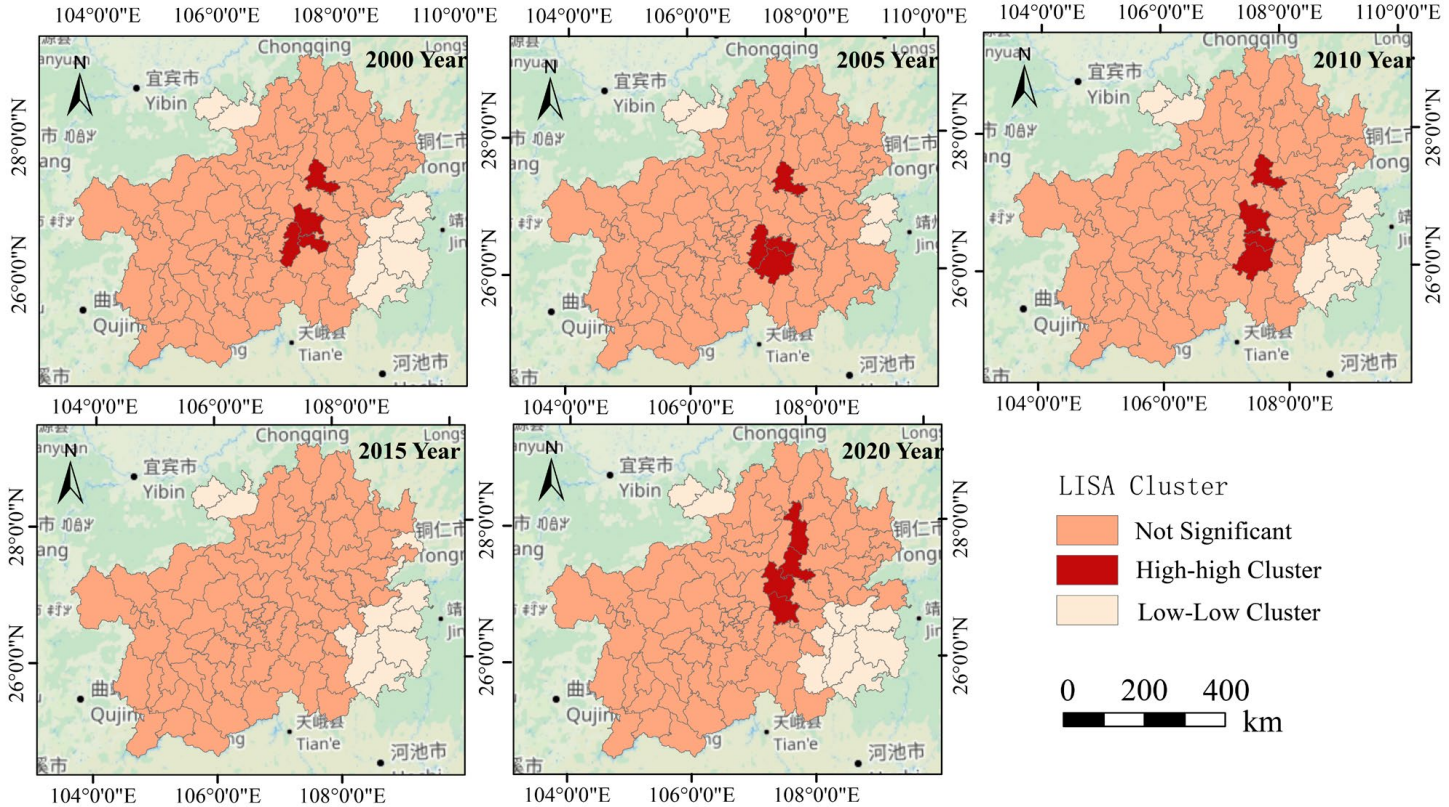
Local autocorrelation clustering distribution of ecosystem health in Guizhou province.

The local autocorrelation cluster maps of EHI in Guizhou Province for the years 2000, 2005, 2010, 2015, and 2020 are shown in Figure 5. It can be observed that during the study period, most areas in Guizhou Province did not exhibit a significant clustering characteristic for EHI. “H‐H clusters” (high‐high clusters) are mainly distributed in the northern part of Qiannan Prefecture and the southern part of Zunyi, including counties and cities such as Yuqing, Puding, and Duyun; “L‐L clusters” (low‐low clusters) are distributed in the eastern part of Qiandongnan Prefecture and the northwestern part of Zunyi, including Chishui, Xishui, Tianzhu, and Jinping, indicating that the ecological environment in these areas is poor, and the ecological environment in adjacent areas is also poor. Areas without significant clustering characteristics are widely and sparsely distributed.

### 4.3 Analysis of Driving Factors of Ecosystem Health

#### 4.3.1 Selection and Identification of Influencing Factors

To explore the driving factors of ecosystem health changes in Guizhou Province, based on the overview of the study area and data availability, DEM (Digital Elevation Model), SLOPE (slope), PRE (precipitation), TEM (temperature), RHU (relative humidity), and FVC (fraction of vegetation coverage) as natural factors, and GDP (Gross Domestic Product), NLI (nighttime light intensity), POP (population), and LUI (land use intensity) as human activity factors were selected for the years 2000, 2005, 2010, 2015, and 2020. A GTWR (Geographically and Temporally Weighted Regression) model was constructed to further analyze the driving factors of ecosystem health changes in Guizhou Province. To avoid the issue of multicollinearity among the initial factors and to achieve effective dimensionality reduction, the 9 initial factors were applied to OLS (Ordinary Least Squares) classical linear regression. Based on the regression results, factors with a variance inflation factor (VIF) greater than 7.5, such as DEM and POP, were excluded. The remaining 6 factors were analyzed with Guizhou's EHI (Ecosystem Health Index) using the GTWR model.

#### 4.3.2 Based on GTWR'S Analysis of the Spatiotemporal Heterogeneity of Influencing Factors

**FIGURE 6:**
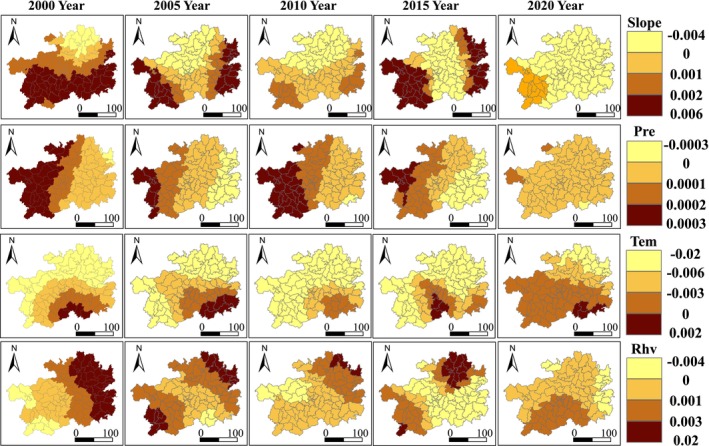
Spatiotemporal distribution of regression coefficients for ecosystem health influencing factors (Slope, Pre, Tem, Rhv) in Guizhou Province.

**FIGURE 7:**
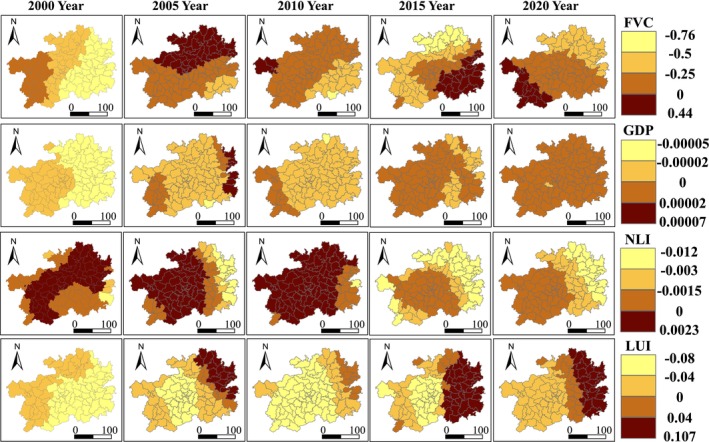
Spatiotemporal Distribution of Regression Coefficients for Ecosystem Health Influencing Factors (FVC, GDP, NLI, LUI) in Guizhou Province.

Based on the GTWR model, a spatiotemporal geographically weighted analysis was conducted between various factors and EHI. The regression coefficients were divided into four levels. Figures 6 and 7 display the spatiotemporal distribution characteristics of the regression coefficients for natural and anthropogenic factors. Overall, different influencing factors have spatiotemporal differences in their impact on the ecosystem health of Guizhou Province. Among them, PRE (precipitation), RHU (relative humidity), and NLI (nighttime light intensity) from 2000 to 2015 mainly have a positive driving effect on the ecosystem health of Guizhou Province, while SLOPE (slope), TEM (temperature), FVC (fraction of vegetation coverage), GDP (Gross Domestic Product), and LUI (land use intensity) mainly have a negative driving effect. TEM, FVC, and LUI have higher absolute values of regression coefficients compared to other factors; thus, these three factors are identified as the dominant factors affecting the ecosystem health of Guizhou Province, and their impact on EHI is analyzed in detail.

The impact of temperature on the ecosystem health of Guizhou Province is mainly manifested as a negative driving effect, which is characterized by a decrease in EHI as TEM increases. In most areas of Guizhou Province, the regression coefficients of EHI and TEM are less than 0, and the overall southern region has higher regression coefficients than the northern region. The high‐value areas are mainly distributed in the southern part of Qiannan Prefecture, and over time, this positive driving effect gradually shifts eastward. Low‐value areas are widely and sparsely distributed. Looking at the trend of change, the area of significant low‐value regression coefficients gradually decreases, indicating that the negative driving effect of temperature on EHI in this region is weakening.

From the spatiotemporal distribution of the regression coefficients between FVC (Fraction of Vegetation Cover) and EHI (Ecosystem Health Index) as shown in Figure 7, it can be observed that in most areas of Guizhou Province, the regression coefficients of EHI with respect to FVC are less than 0, indicating that FVC has a predominantly negative driving effect on ecosystem health in Guizhou Province. This is characterized by a decrease in EHI as FVC increases. The high‐value distribution areas shifted from Zunyi City in 2005 to the western part of Bijie City in 2010, to Qiandongnan Prefecture in 2015, and to the western part of Guizhou Southwest Prefecture and Liupanshui City in 2020. The low‐value areas do not show a clear pattern.

The impact of land use intensity (LUI) on ecosystem health in Guizhou Province is also primarily a negative driving effect, with regression coefficients generally showing a trend of being higher in the eastern region than in the western and central regions. Overall, from 2015 to 2020, the eastern region of Guizhou Province mainly had a positive driving effect on ecosystem health, while from 2000 to 2015, the central region had a stronger negative driving effect. Looking at the changes over time, the negative driving effect of LUI on ecosystem health has weakened.

## 5 Discussion and Conclusion

### 5.1 Discussion

As a typical karst rocky desertification region, Guizhou Province's ecosystem health is jointly influenced by natural factors (e.g., climate, geomorphology) and human activities (e.g., land use, urbanization) (Xiao et al. 2024; Das et al. 2021). This study, based on the PSR framework and GTWR model, reveals an overall upward trend in ecosystem health, albeit with significant spatial heterogeneity. Certain areas (such as Qiandongnan Prefecture and Chishui City) still face considerable ecological pressures. While ecological restoration efforts have achieved notable results, challenges persist. Recent policies like the Grain‐for‐Green Program and comprehensive rocky desertification control have significantly improved ecosystem health in Guizhou. However, some regions remain at risk of ecological degradation due to geological fragility, overexploitation, or oversimplified vegetation restoration approaches (Chapin III et al. 2000). Climate change exerts dual effects on ecosystem health: precipitation and humidity positively contribute to ecological health, whereas rising temperatures may exacerbate drought stress. Future attention should focus on the long‐term impacts of global warming on hydrological processes in karst areas. The positive correlation observed with nighttime light data suggests potential environmental governance improvements accompanying urbanization. Nevertheless, intensive agriculture and urban expansion continue to pose major threats, necessitating optimized land use practices.

Based on the research findings and the characteristics of rocky desertification in Guizhou Province, we propose the following integrated ecological conservation and sustainable development strategies: (1) Optimize vegetation restoration by promoting mixed forests with native species to enhance ecosystem resilience and water conservation, while implementing differentiated rehabilitation approaches combining natural regeneration and artificial restoration in critical areas like Qiandongnan Prefecture and Chishui City; (2) Strengthen land‐use regulation through ecological redlines to control development in sensitive areas and promote ecological agriculture to reduce soil erosion; (3) Enhance climate adaptation by establishing drought‐resistant vegetation systems and improving water infrastructure in precipitation‐variable regions; (4) Establish cross‐county ecological compensation mechanisms and strengthen monitoring systems using remote sensing and ground‐based methods for dynamic assessment and adaptive management.

### 5.2 Conclusions

Based on the PSR framework and GTWR model, this study reveals the spatiotemporal evolution patterns and driving mechanisms of ecosystem health in Guizhou Province from 2000 to 2020. The main conclusions are as follows: The overall ecosystem health has improved, but significant spatial heterogeneity exists, with key areas such as Qiandongnan Prefecture and Chishui City still requiring focused governance. Climatic factors (precipitation, temperature) and human activities (land use, urbanization) are the primary drivers, necessitating differentiated regulatory measures. Future ecological management should integrate vegetation optimization, land use control, and climate adaptation. This study established an ecosystem health assessment system for karst rocky desertification areas based on the PSR model, but there remains room for improvement in the following aspects: Firstly, when applying the nationwide VCI to calculate ecosystem service values, the distinctive ecological characteristics of the study area were not fully accounted for. Secondly, constrained by data availability, technical limitations such as insufficient spatial resolution and incomplete temporal sequences persist. Additionally, the translation mechanism of research findings into policy implementation still requires refinement. To address these issues, subsequent research should prioritize the following initiatives: Conduct parameter localization by adjusting benchmark unit prices using local grain yield and economic data from Guizhou Province, while integrating multi‐source high‐resolution remote sensing data. It is also essential to couple process‐based mechanism models with socio‐economic dynamic models and establish a multi‐stakeholder policy coordination framework. These improvements will provide more precise decision‐making support for ecosystem governance in karst regions, while improving ecological compensation mechanisms to promote sustainable development in karst regions.

The rocky desertification control in Guizhou Province has achieved phased success, but long‐term dynamic monitoring and policy optimization are still required. This study provides a scientific basis for the fragile karst ecological zones. Future research could further optimize ecological restoration pathways by incorporating scenario simulations, thereby supporting regional ecological civilization construction.

## Author Contributions

**Beibei Zhang:** conceptualization (equal), data curation (equal), formal analysis (equal), writing – original draft (equal), writing – review and editing (equal). **Zhongfa Zhou:** funding acquisition (equal), resources (equal), software (equal).

## Conflicts of Interest

The authors declare no conflicts of interest.

## Supporting information


Data S1.

## Data Availability

All the required data are uploaded as Supporting Information.
